# Early adopters of 6-month levofloxacin as rifampicin-resistant tuberculosis preventive treatment regimen in the WHO European Region, 2023

**DOI:** 10.2807/1560-7917.ES.2025.30.30.2500210

**Published:** 2025-07-31

**Authors:** Irina Felker, Alexandra Solovyeva, Ana Ciobanu, Araksya Hovhannesyan, Dennis Falzon, Andrei Dadu

**Affiliations:** 1 Novosibirsk State Medical University, Department of Phthisiopulmonology, Novosibirsk, Russian Federation; 2 European TB Programme, World Health Organization Regional Office for Europe, Copenhagen, Denmark; 3 Global Programme on Tuberculosis & Lung Health, World Health Organization, Geneva, Switzerland

**Keywords:** tuberculosis, preventive treatment, levofloxacin, drug-resistant TB, WHO European Region

## Abstract

Tuberculosis (TB) preventive treatment (TPT) is crucial for preventing infection with *Mycobacterium tuberculosis* from progressing to TB disease, especially among people in high-risk groups. The expansion of novel TPT regimens for drug-susceptible TB is a notable advancement in TB care. However, managing contacts of drug-resistant TB patients remains a major challenge, particularly in Eastern Europe and Central Asia. In 2020, the World Health Organization (WHO) recommended TPT for high-risk household contacts of multidrug-resistant (MDR) or rifampicin-resistant (RR) TB patients; this was further reinforced in 2024 with a recommendation of a 6-month levofloxacin (6-Lfx) regimen. This Perspective discusses the early adoption of 6-Lfx for MDR/RR-TPT in the WHO European Region. In 2023, 38 of 53 WHO European Region countries reported on 6-Lfx use, with only eight confirming its use for MDR-TB contact persons. Accelerating the adoption of the 6-Lfx regimen and other evidence-backed TPT regimens is crucial for achieving TB elimination in the WHO European Region. Addressing challenges such as slow uptake of the recommendations, low awareness in affected communities and resource shortages are essential for success.

## Background

Tuberculosis (TB) preventive treatment (TPT) is the main healthcare intervention available to reduce the risk of TB infection progressing to TB disease [1]. Mathematical impact modelling suggests that systematic screening for TB and TPT have a crucial role within the full cascade of TB care implementation needed to advance towards TB elimination at the regional level [2]. Tuberculosis (TB) preventive treatment guidelines published by the World Health Organization (WHO) in March 2020 and September 2024 highlight the importance of appropriate programmatic management of infection with *Mycobacterium tuberculosis* in accomplishing the targets of the End TB Strategy [1,3,4]. These guidelines outline the key interventions for TB elimination [5,6]. They focus in particular on population groups at the highest risk of infection, and progression from infection to disease. These groups include individuals of all ages who have been in contact with patients with pulmonary TB, people living with HIV and other populations with clinical and social risk factors.

The expansion of novel TPT regimens for individuals exposed to drug-susceptible *M. tuberculosis* represents a breakthrough in strengthening the full cascade of TB care. However, this progress does not address the critical issue of managing contacts exposed to multidrug-resistant or rifampicin-resistant TB (MDR/RR-TB); it is likely that standard TPT with isoniazid and/or rifamycin in these circumstances would not be effective. In 2023, the average prevalence of MDR/RR-TB among new, bacteriologically- confirmed pulmonary TB cases in the WHO European Region was 20%, with higher levels in Eastern Europe and Central Asia [7]. This presents a substantial impediment to achieving the End TB Strategy goals by 2030 in the WHO European Region.

Given the high occurrence of MDR/RR-TB in the WHO European Region, it is critical to address the challenge of TPT among people of all ages who have been in contact with MDR/RR-TB patients, either through programmatic implementation or through operational research. In 2020, the WHO conditionally recommended TPT for high-risk household contacts of individuals with MDR/RR-TB based on observational data [1]. This recommendation was further reinforced in September 2024, when the 6-month levofloxacin (6-Lfx) as TPT was strongly recommended based on evidence from randomised control trials [3]. Although the two trials did not show a statistically significant reduction in TB incidence in MDR/RR-TB contacts compared with controls, a similar large effect was noted independently in both [8,9]. This, combined with a good safety profile and the high likelihood that benefits outweighed potential harms, supported a strong recommendation for the use of 6-Lfx in programmatic settings. In this Perspective, we assess the practice of early implementation of the 6-Lfx TPT regimen in countries of the WHO European Region.

## Reported implementation

Data were extracted from the WHO’s global TB database in February 2025, which contains country reports collected annually to assess progress of interventions on TB prevention and care [10]. We analysed only the single variable indicating whether 6-Lfx was used in contact persons of MDR/RR-TB patients. We defined ‘early adopters’ as countries that adopted 6-Lfx by 2023, the year before WHO issued its strong recommendation. Data on the use of the 6-Lfx TPT regimen started in in 2023, so earlier data were not available for analysis. Although the variable in the database was formulated as ‘use of the 6-Lfx regimen at least once’, the countries using the regimen do so either on a programmatic scale under an executive order of the ministry of health (most common practice) or on a pilot scale under operational research conditions upon endorsement by a local ethical review committee. We computed the number of early adopters expressed as a fraction of all countries that reported data related to TPT.

In 2023, 38 of 53 WHO European Region countries answered the question about the use of 6-Lfx among contacts of people diagnosed with MDR/RR-TB. Of these 38 countries, only eight confirmed that 6-Lfx has been used at least once in contacts exposed to MDR/RR-TB, namely Georgia, Kazakhstan, Portugal, Serbia, Slovakia, Türkiye, Ukraine and Uzbekistan. Additional 21 countries reported not using the 6-Lfx regimen, while for nine countries it was reported that they do not know whether the regimen is used (Figure). Countries that used 6-Lfx had widely differing prevalences of MDR/RR-TB among newly diagnosed patients, ranging from 0.87% in Portugal to 33.36% in Kazakhstan, and among previously treated patients, which ranged from 6.07% (Serbia) to 52.78% (Kazakhstan) [7].

**Figure:**
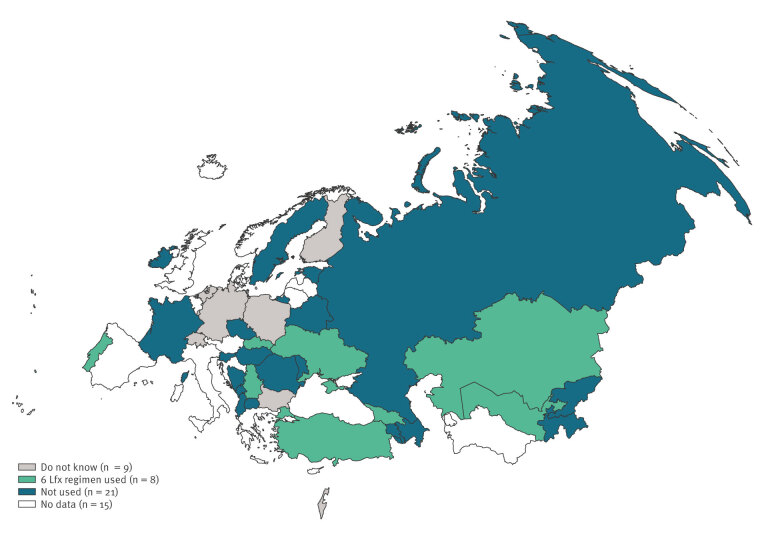
Use of a 6-month levofloxacin regimen for tuberculosis preventive treatment, among contacts of MDR/RR TB cases, WHO European Region, 2023 (n = 53 countries)

WHO: World Health Organization; 6-Lfx: 6-month levofloxacin regimen.

These data suggest that several countries had an interest in using 6-Lfx even before the WHO issued a strong recommendation for its use. However, additional efforts are needed to accelerate the scale-up of the regimen and speed up the implementation of other safe TPT regimens that are shorter and can be used despite rifampicin and isoniazid resistance.

## Challenges for implementation

The WHO interactions with the TB national counterparts in the countries in 2022–24 during in-person and virtual meetings, as well as country programme reviews, showed that challenges remain in implementing TPT for MDR/RR-TB. These include limited data on its uptake, completion rates and effectiveness across different populations and geographical areas, as well as factors associated with treatment-related toxicities. Furthermore, a substantial proportion of MDR/RR-TB cases globally are fluoroquinolone-resistant (defined as pre-extensively drug-resistant TB; pre-XDR-TB), with an estimated 31.1% among MDR/RR-TB cases in the WHO European Region in 2023 [10]. In case of contact with pre-XDR-TB patients, 6-Lfx TPT is not expected to be effective. There is currently no high certainty evidence to support recommendations on TPT regimens for contacts of individuals with pre-XDR-TB. Provisional results indicate that the use of a 3-month bedaquiline regimen (3Bdq) for TPT among contact persons exposed to pre-XDR-TB is feasible, effective and safe [11].

## Regional response

In 2023, the TB programmes at the WHO European Region and WHO headquarters, with support from donors and technical partners, organised a face-to-face workshop specifically planning on TB prevention and systematic screening [12]. This interaction served as a follow up on the regional meeting of national TB counterparts on the Implementation of the Tuberculosis Action Plan for the WHO European Region 2023–30 for High-Priority Countries to End TB by 2030. To enhance TB screening and expand TPT among at-risk populations in the WHO European Region, the European Tuberculosis Programme established an operational initiative, The European Prevention and Systematic Screening Initiative to End TB (known as PASS to End TB) in the WHO European Region by 2030. These efforts, packaged into an operational framework, the ‘PASS/Accelerator’, are centred on intensive collaboration within the National TB Programme (NTP), linking TB prevention and screening efforts with diagnosis, treatment and care for people belonging to vulnerable and at-risk populations. Modus operandi also ensures intersectoral collaboration beyond the TB programme, by engaging counterparts from key health system components such as governance, financing, medicines, workforce and information. The operational initiative utilises the newly established PASS network, which is comprised of NTP-nominated focal points, WHO temporary advisors, technical partners and the WHO secretariat, to provide structured and catalytic support to countries. This support aims to guide the implementation of service delivery standards through policy adaptation, capacity building, operational research and impact monitoring. The policies enhancement is driven by adaptation of WHO recommendations to national contexts, using standardised tools, such as cascade of care data analysis and mathematical modelling (screntb2.0) [13]. Capacity strengthening thought a rollout of cascade of trainings to strengthen the collaboration among phthisiopulmonologists, family doctors, and public health epidemiologists. Meanwhile, research and innovation drives specifically preventive treatment for M/XDR-TB under operational research conditions (PTOR), ensuring universal coverage for household and close contacts. Response and impact monitoring evaluates outcomes of TB prevention and screening efforts, emphasising benchmarks and cascade of care analysis along patient pathways. Inclusive engagement with civil society and affected communities is also encouraged [14,15].

To facilitate the generation of harmonised data across various implementation settings, WHO’s European TB programme has developed a master protocol for the implementation of drug-resistant TB preventive treatment under operational research conditions, known as PTOR. The primary objectives of PTOR are to understand the real-world effectiveness and safety of DR-TB preventive treatment in participating European countries and to evaluate the entire cascade of care from screening to treatment completion. Regimens considered include: 6-month daily levofloxacin or moxifloxacin, 3-or 6-month bedaquiline or delamanid respectively, for FQ-resistant TB. The choice of preventive treatment regimen for contact persons invited to enrol is driven by factors such as the intensity of contact, the index patient's resistance profile (specifically bacteriological confirmed MDR/RR-TB or pre-XDR-TB), and the confirmation of infection with *M. tuberculosis* using tests like interferon-gamma release assay (IGRA), of new TB antigen-based skin tests (TBST). Clinical observation for 2 years, during which the contact is monitored for signs or symptoms of TB, will follow the preventive treatment onset. Endorsed by the WHO Ethics Review Committee, this protocol serves as a foundation for country-context adaptation and enables studies to be conducted using a standardised methodology. In preparation for PTOR implementation, a country readiness assessment was conducted in Belarus, Georgia, Kazakhstan, Tajikistan, Ukraine, and Uzbekistan, while the responses of remaining Eastern Europe and Central Asia countries are pending. Following the readiness assessment, the planned timeline for the PTOR includes: (i) a formal nomination of principal countries investigators, followed by a series of country dedicated workshops on adapting the protocol to country contexts; (ii) first enrolments in January 2026 with anonymised data hosted by WHO on the REDCap platform; and (iii) preliminary and final results anticipated by end of June 2027 and early January 2029, respectively.

The accelerated implementation of the PTOR protocol in settings with a high prevalence of MDR/RR-TB and pre-XDR-TB should help to address the existing research gap on the effectiveness and safety of TPT regimens for contacts of individuals with different patterns of drug-resistant TB. The WHO is currently analysing the association of use of 6-Lfx regiment and countries’ TB context and success factors for its adoption for the pragmatic use via a survey, with results expected in early 2026. Such evidence will be crucial for informing policy decisions and optimising preventive strategies.

## Conclusion

In 2023, 38 of 53 WHO European Region countries reported on the use of 6-Lfx, with only eight countries confirming its use for MDR-TB contacts. Considering that this occurred before the WHO’s strong recommendation for the use of 6-Lfx was published in February 2024, we expect that its use will increase in future years. Accelerating the adoption of 6-Lfx regimen and other alternative drug-resistant TB preventive treatment regimens is crucial for achieving TB elimination in the WHO European Region. Addressing challenges such as slow uptake of recommendations, low community awareness, and resource shortages are essential for success.

## Data Availability

The insights presented in this perspective article are based on a synthesis of publicly available data and official reports submitted by the countries to WHO, among them the Global TB database. All sources are fully referenced within the manuscript. No new datasets were generated for this publication.

## Disclaimer

The views and opinions expressed in this paper are those of the authors and not necessarily the views and decisions or policies of the World Health Organization, Novosibirsk State Medical University, Department of Phthisiopulmonology, Novosibirsk, Russian Federation.

The designations used and the presentation of the material in this publication do not imply the expression of any opinion whatsoever on the part of WHO the legal status of any country, territory, city or area, or of its authorities, nor concerning the delimitation of its frontiers or boundaries.
